# Effects of pre-eclampsia/eclampsia on platelet parameters in small for gestational age preterm infants

**DOI:** 10.3389/fped.2025.1622610

**Published:** 2025-08-26

**Authors:** Huiling Huang, Jing Zhao, Jichong Huang, Yuan Ai, Tingting Zhu

**Affiliations:** ^1^ Department of Pediatrics, West China Second University Hospital, Sichuan University, Chengdu, Sichuan, China; ^2^ Medical College Shantou University, Shantou, Guangdong, China; ^3^ Key Laboratory of Obstetric & Gynecologic and Pediatric Diseases and Birth Defects of Ministry of Education, Sichuan University, Chengdu, Sichuan, China

**Keywords:** pre-eclampsia, eclampsia, platelet, thrombocytopenia, small for gestational age

## Abstract

**Objectives:**

To investigate whether pre-eclampsia/eclampsia (PE/E) alters platelet parameters, including platelet count (PLT), mean platelet volume (MPV), and platelet distribution width (PDW), in small for gestational age (SGA) infants.

**Methods:**

We enrolled 245 SGA preterm infants between 2020 and 2023. They were grouped according to their mother's PE/E status. We assessed the PLT, MPV, PDW, and prevelence of thrombocytopenia during the first 30 days after birth. Groups were compared using either *χ*2 test or Fisher's exact test.

**Results:**

SGA neonates born to mothers with PE/E had a lower PLT on Day 7 than those born to mothers without PE/E (*P* = 0.0211). PDW and MPV values gradually increased for the first several days, and eventually stabilized around the first week. There was no statistical difference in MPV, PDW, or thrombocytopenia prevalence between SGA neonates between born to mothers with PE/E and those born to mothers without PE/E.

**Conclusions:**

Evidence to support an association between PE/E and platelets parameter in neonates is limited. SGA may be the real reason for alterations in platelet parameters in neonates.

## Introduction

Small for gestational age (SGA) infants are those whose birth weight (BW) is below the 10th percentile for gestational age (GA) and sex compared to a given reference population (1). Global estimates for 2020 suggest that 13.4 million live births were preterm, with rates over the past decade remaining static, and 23.4 million were SGA (2). There is a a higher proportion of SGA infants among term and preterm infant in low-income countries compared to high income countries (3, 4). The prevalence of preterm SGA infants in Africa increased up to 12.3% (4). Children born SGA have higher perinatal morbidity and mortality compared to appropriate-for-gestational-age (AGA) infants, and represent a major challenge in perinatal management.

Previous studies have reported that SGA infants have low peripheral white blood cell, absolute neutrophil, and platelet counts (PLTs) in the first few days after birth (5–7). Takeshita et al. (7) reported that PLTs were significantly lower in the SGA group than in the non-SGA group at the time of the lowest PLT within 72 h of birth. Approximately 30% of SGA neonates develop thrombocytopenia during the first week after birth (6), which is associated with high mortality (7). However, the cause of these alterations in the blood cell counts of SGA infants remains unknown.

Preeclampsia/eclampsia (PE/E) is among the most common complications of pregnancy and the leading cause of premature delivery and SGA in infants. Preeclampsia is characterized by new-onset hypertension and is often accompanied by proteinuria (8). Eclampsia represents a severe progression of preeclampsia and is defined by the occurrence of seizures in a patient who has preeclampsia (8). The increasing prevalence of hypertension poses challenges for both mothers and neonates. Up to 15% of mothers have hypertensive disorders during pregnancy (9). In China's National Maternal Near-Miss Surveillance System, 2.27% of pregnant women had superimposed preeclampsia, and 50.17% had pre-eclampsia or eclampsia from 2012 to 2020 (10). PE/E is closely related to neonatal death and hypothesized to induce a lower PLT in the offspring (11).

Therefore, in this retrospectively study, we investigated the effect of PE/E on platelet parameters in SGA infants. We aimed to investigate (1) platelet parameters, alterations in SGA infants during the first 30 days after birth; (2) whether PE/E influenced their platelet parameters; and (3) whether it increased their risk of thrombocytopenia in SGA infants.

## Methods

The level III neonatal intensive care unit (NICU) of West China Second University Hospital of Sichuan University is the largest neonatal medical unit in Western China. The demographic, clinical, and laboratory data of neonates and their mothers were retrieved from its electronic medical record system. All preterm neonates (GA <37 weeks) with SGA admitted to the NICU within 24 h after birth between November 1st, 2020 and January 1st, 2023 were retrospectively enrolled. Informed written consent was obtained from the parents after the nature of the study was fully explained to them. This study was approved by the Ethics Committee of Sichuan University. All research processes were conducted according to the relevant guidelines and regulations.

SGA was diagnosed based on normative values (12). PE/E was classified based on the criteria of the American College of Obstetricians & Gynecologists (13). Preterm SGA neonates were excluded due to lack of complete blood count (CBC), death within 24 h after admission to the NICU, and the presence of other diseases associated with platelet reduction such as congenital malformation, perinatal asphyxia (Apgar score at 1 min less than 4), early-onset sepsis (defined as isolation of a pathogenic organism from blood and/or CSF culture obtained within 3 days after infant birth), disseminated intravascular coagulation, and immune-mediated thrombocytopenia.

Neonates were grouped according to their mother's PE/E status. We collected characteristic data including maternal age, antenatal steroid usage, sex, BW, GA, Apgar score, mode of delivery, and clinical conditions. Neonatal outcome included mortality, duration of mechanical ventilation, length of hospitalization, hemodynamically significant patent ductus arteriosus, necrotizing enterocolitis (Bell's stage ≥II), bronchopulmonary dysplasia (BPD, defined as the use of O2 at 36 wk postmenstrual age), severe intracranial hemorrhage (IVH, ≥grade 3) and retinopathy of premature infants (ROP, ≥grade 3). Platelets parameters of venous blood samples were measured using automated analyzers at the Clinical Laboratory of West China Second University Hospital. We extracted each neonate's CBC from the laboratory database, if more than one CBC was conducted on the same day, the lowest PLT value was selected. We calculated the mean platelet volume (MPV) and platelet distribution width (PDW). We tried to demonstrate the value changes of PLT, PDW and MPV in preterm SGA after birth. We calculated the mean and various percentile values of PLT, PDW and MPV during first week and different time interval after first week.

The data were compared between the PE/E group and non-PE/E groups. Continuous variables were expressed as the mean ± SD (standard deviation) or median with the interquartile range, based on the data distribution, and were compared using the Student's *t*-test or Mann–Whitney *U* test, respectively. We described the categorical variables using numbers and percentages (%), and compared them with either *χ*^2^ test or Fisher's exact test. All statistical analyses were performed using IBM SPSS Statistics (Windows, version 25.0.).

## Results

**Figure 1:**
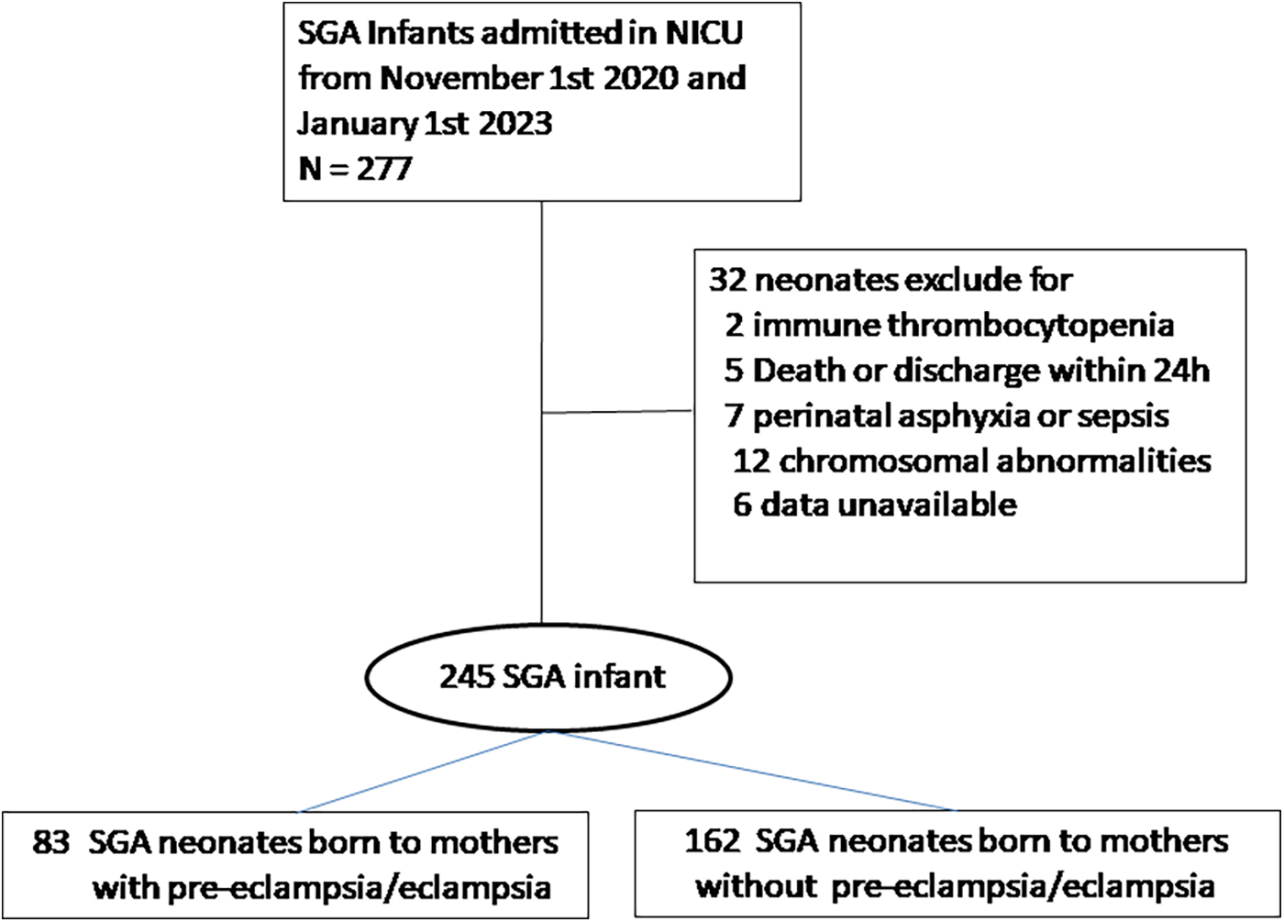
Study flowchart showing numbers of neonates enrolled.

During the study period, 277 preterm SGA neonates were admitted to our NICUs; 32 were excluded because of congenital malformations, early-onset sepsis, severe neonatal asphyxia, death, or discharge 24 h after birth. Therefore, a total of 245 SGA neonates were included in the analysis (Figure 1).

The baseline characteristics of mothers and neonates, including BW, GA, sex, cesarean delivery rate, antenatal steroid use, maternal diabetes and intrahepatic cholestasis of pregnancy are shown in Table 1. The mean GA and BW in the PE/E group were 33.2 weeks and 1,449.8 gram. The mean GA and BW in the non-PE/E group were 34.1 weeks and 1,593.4 gram. The proportion of infants with a BW <1,500 g was significantly higher in the PE/E group. More infants in the PE/E group were administered antenatal steroids. The mean PLT, MPV, and PDW on the first day did not significantly differ between the two groups.

**Table 1:** Maternal and infant clinical data of SGA preterm infants.

Characteristics	PE/E (*n* = 83)	no PE/E (*n* = 162)	*p* value
GA (weeks), mean (SD)	33.2 (1.9)	34.1 (2.0)	0.0009
GA group (%)			
<32 w	16 (19.3)	15 (9.3)	0.0406
32 ≤ 34 w	26 (31.3)	31 (19.1)	0.0383
34 ≤ 37 w	41 (49.4)	116 (71.6)	0.0007
BW (gm), mean (SD)	1,449.8 (326.0)	1,593.4 (351.3)	0.0023
BW group (%)			
<1,500 g	44 (53.0)	60 (37.0)	0.0203
1,500–2,000 g	37 (44.6)	89 (54.9)	0.1386
>2,000 g	2 (2.4)	13 (8.1)	0.0969
Male (%)	36 (43.4)	78 (48.1)	0.5012
Singleton pregnancy (%)	54 (65.1)	61 (37.7)	<0.0001
Cesarean delivery (yes, %)	2 (2.4)	13 (8.0)	0.0969
Antenatal steroid use (yes, %)	67 (80.7)	93 (57.4)	0.0004
Surfactant use (yes, %)	13 (15.7)	14 (8.6)	0.1300
Invasive ventilation (yes, %)	13 (15.7)	19 (11.7)	0.4253
Maternal ICP (yes, %)	8 (9.6)	18 (11.1)	0.8285
Maternal diabetes (yes, %)	14 (16.9)	40 (24.7)	0.1937
Platelet count in first day (10^9^/L), mean (SD)	211.1 (77.7)	210.3 (64.0)	0.8609
MPV in first day (fL), mean (SD)	10.2 (0.9)	10.2 (0.8)	0.8961
PDW in first day (fL), mean (SD)	11.5 (1.9)	11.5 (2.2)	0.8002

PE/E, preeclampsia/eclampsia; SGA, small for gestational age infants; GA, gestational age; BW, birth weight; w, weeks; gm, grammage; ICP, intrahepatic cholestasis of pregnancy; SD, standard deviation; PLT, platelet count; MPV, mean platelet volume; PDW, platelet distribution width.

Table 2 shows the neonatal outcomes of the study population. Our results showed that SGA infants born to women with non-PE/E had longer hospital stay. This could be due to neonatal necrotizing enterocolitis (NEC), and patients with BPD in the PE/E group had more severe diseases and required longer treatment time. There were no significant differences in any of the other neonatal outcomes, including NEC, BPP, severe ROP, severe IVH, blood transfusion, and mortality between the two groups.

**Table 2:** Comparison of neonatal outcome between the groups of maternal preeclampsia/eclampsia in preterm SGA.

Outcome	PE/E (*n* = 83)	no PE/E (*n* = 162)	*p* value
Length of hospital (day, mean, SD)	26.8 (11.9)	35.5 (20.6)	0.0276
Length of invasive ventilation (day, mean, SD)	0.7 (2.1)	0.7 (2.8)	0.9904
Length of non-invasive ventilation (day, mean, SD)	6.1 (9.4)	3.3 (8.0)	0.0193
Transfusion (yes, %)	12 (14.4)	17 (10.5)	0.4043
NEC (yes, %)	5 (6.0)	11 (6.8)	1.0000
BPD (yes, %)	3 (3.6)	7 (4.3)	1.0000
Severe ROP (yes, %)	3 (3.6)	7 (4.3)	1.0000
Severe IVH (yes, %)	1 (1.2)	0 (0)	0.3388
hs PDA (yes, %)	4 (4.8)	4 (2.5)	0.4490
Mortality (yes, %)	1 (1.2)	1 (0.6)	1.0000

PE/E, preeclampsia/eclampsia; SGA, small for gestational age infants; SD, standard deviation; BPD, bronchopulmonary dysplasia; IVH, intraventricular hemorrhage; ROP, retinopathy of prematurity; NEC, necrotizing enterocolitis; hs PDA, hemodynamically significant patent ductus arteriosus.

**Figure 2:**
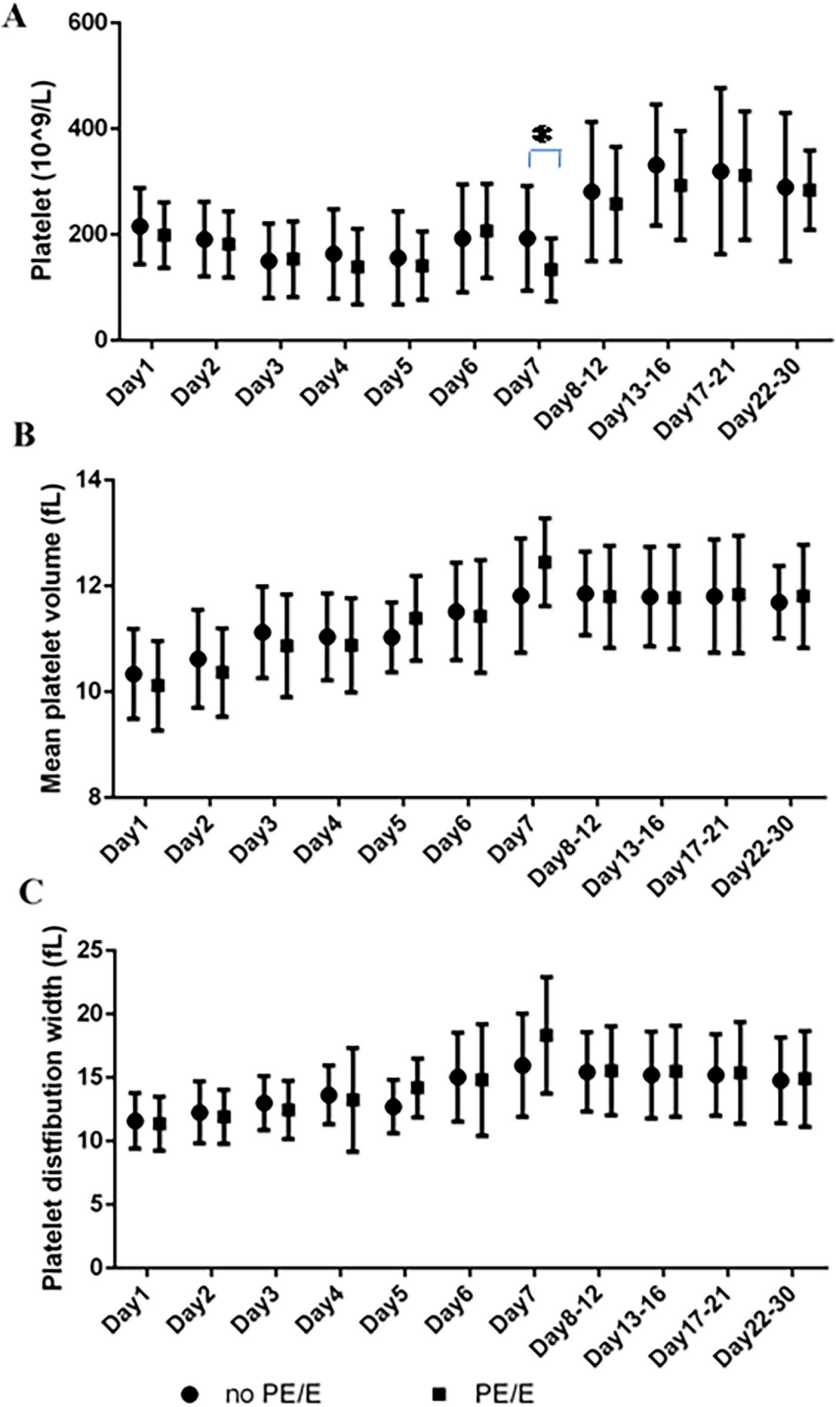
Platelets parameters at the various time points in maternal PE/E group and control group during their first 30 days. **(A)** Platelet count; **(B)** mean platelet volume; **(C)** platelet distribution width. (*P = 0.0211; On Day 7, SGA neonates born to mothers with PE/E had lower PLTs than those born to mothers without PE/E).

The PDW and MPV values of the two groups are shown in Figure 2. The ranges for PDW and MPV in the neonates during their first month (5th to 95th percentile limits) were shown in Supplementary File 1. The PDW and MPV values did not differ between PE/E and non-PE/E group (*P* > 0.05); they both gradually increased during the first week. The PDW values will eventually stabilized on days 6–7, and those of MPV on day 5–7.

Lower PLTs were observed in the first week (See Supplementary File 2). On Day 7, SGA neonates in the PE/E group had lower PLTs (mean 133 × 10^9^/L) than those in the non- PE/E (*P* = 0.0211).

**Figure 3:**
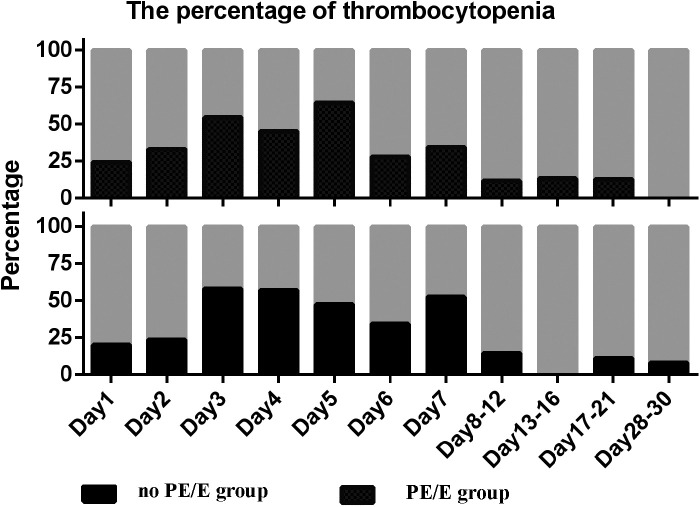
Percentage of thrombocytopenia in small for gestational age infants with or without maternal preeclampsia/eclampsia during the study period. Thrombocytopenia defined as PLT less than 150 × 10^9^/L; PE/E, maternal preeclampsia/eclampsia group; no PE/E, no maternal preeclampsia/eclampsia group.

There were no significant differences in the prevalence of thrombocytopenia (<150 × 10^9^/L) in SGA neonates during the first month between the two groups (*P* < 0.05). The prevalence in both groups was >20% during the first week, then peaked at 58% and 64% over the next 3 weeks in non-PE/Eand PE/E group, respectively (Figure 3).

## Discussion

Our study showed that the PLT, MPV, and PDW decreased in SGA infants in both the PE/E and non-PE/E groups, throughout the first week after birth. PE/E did not influence the infant's first PLT or increase the prevalence of thrombocytopenia and did not affect PDW and MPV.

Studies on PDW and MPV in SGA neonates are scarce. Wasiluk et al. (14) used blood samples collected from the umbilical artery to compare 61 full-term SGA newborns with 71 full-term AGA newborns. MPV was higher in SGA newborns (8.25 fl) as compared with AGA newborns (7.84 fl; *P* = 0.008), and while the PDW was nearly identical between groups. Go et al. (15) explored the relationship between perinatal factors and platelet parameters on the first day of life. PDW and MPV did not differ between the SGA and non-SGA groups in late preterm or term neonates. However, the PDW and MPV results in these studies were only compared at a single time point. Despite our small sample size, our study revealed the changes in PDW and MPV in preterm SGA neonates during the 30 days after birth. PDW and MPV values in the early days after birth were lower than those at 1 week after birth (*P* < 0.05 day 7 vs. day 1), they then stabilized with only slight variations over the next 3 weeks. To the best of our knowledge, no study has investigated the effects of PE/E on MPV or PDW in SGA neonates. One study conducted by Elgari et al. (16) reported that MPV in umbilical cord blood of newborns was not associated with PE (cord blood of mothers with PE vs. control group, mean = 9.8 ± 0.98 vs. mean = 9.8 ± 1.0). Participants in this study include full-term and preterm babies, and their mean GA of the PE group was 37.4 ± 2.4 weeks. In the present study, we selected only preterm SGA neonates, and compared the results of PDW and MPV for different days after birth. PE/E did not influence PDW or MPV in preterm SGA neonates during the first month. However, more cohort studies with lager sample size are needed to confirm these findings.

Thrombocytopenia is common among SGA neonates; however, the correlation between PLT in neonates and PE/E remains unknown. A study by Delaney et al. focused on thrombocytopenia among extremely premature infants exposed to maternal hypertension (17). Exposed premature infants had a lower absolute PLT and a higher prevalence of PLT <150 × 10^9^/L and <100 × 10^9^/L, at their first CBC. PLTs remained low in infants exposed to maternal hypertension at 2, 32 and 36 weeks post-menstrual age, and at discharge. However, mixed effect linear model demonstrated no association between maternal hypertension and the trend of PLTs after adjusting for SGA status (*β* = −9 × 10^9^/L, *P* = .70). Similarly, Joslyn et al. (18) reported that for infants in the subgroup of hypertension and SGA, the presence of either PE with severe features was not associated with a lower first PLT or an increased risk of a first PLT <150 × 10^9^/L in the first 24 h of life. In our study, the proportion of thrombocytopenia cases during the first month after birth was not significantly different between the PE/E and control groups. The mean PLT in the PE/E group was lower than that in the control group at most time points; however, the difference was not statistically significant (*P* > 0.05). We only observed that on day 7, the PLT in SGA infants born to women with PE/E was lower than non-PE/E group (133.5/59.4 vs. 193.0/99.1, *P* = 0.0211). This cannot rule out the potential effect of PE/E on the PLT in neonates.

The proposed mechanisms by which SGA or PE/E affected platelet parameters lack sufficient evidence. It has been previously postulated that the cause of reduced in platelets in SGA was insufficient thrombopoietin (TPO) production, which impairs megakaryopoietic activity (7, 19–20). Takeshita et al. demonstrated that a decrease in TPO production due to hepatic dysmaturation resulted in thrombocytopenia in SGA model rats (19). In agreement with the results, SGA infants with thrombocytopenia had lower immatureplatelet fraction and serum TPO levels than non-SGA infants with thrombocytopenia (7). Cremer et al. (20) hypothesized that moderate and severe thrombocytopenia in SGA neonates might be due to megakaryopoiesis suppression. This is because, they found a trend toward lower immature fractions, which reflects reduced platelet production, in SGA neonates with thrombocytopenia compared with non-SGA neonates with thrombocytopenia who had an infection. Hypertension during pregnancy results in abnormal trophoblast invasion of the spiral arteries during implantation, resulting in placental perfusion deficiency, causing chronic placental insufficiency and subsequent fetal hypoxia (21). The defective trophoblastic invasion characteristic of this condition can also trigger widespread endothelial damage, leading to alterations in the coagulation process between platelets and endothelial cells (22).

Our study had certain limitations. The main GA of the SGA neonates ranged between 34 and 37 weeks. Notably, smaller GA and lower BW are important indicators of thrombocytopenia. Our study did not examine the effect of PE on platelet parameters in extremely preterm infants. We grouped neonates based on the presence or absence of PE/E in their mothers. We did not assess the effects of different forms of maternal hypertension, such as gestational and chronic hypertension, on neonatal platelet parameters. Whether the differentiation of the hypertensive disorders of pregnancy into its subtypes is crucial for platelet parameters remains unknown. Finally, none of the neonates with thrombocytopenia required platelet transfusion. However, the patient clinical manifestations may be relatively mild; therefore, the impact of PE on platelets could have been masked

Despite these limitations, to our best knowledge, this is the first study to explore the impact of PE/E on platelet parameters in preterm neonates with SGA during the first month after birth. Further studies are needed to investigate whether PE/E affects platelet parameters in neonates or whether SGA is an independent risk factor.

## Data Availability

The raw data supporting the conclusions of this article will be made available by the authors, without undue reservation.
