# Supplementary material for: Effects of pre-eclampsia/eclampsia on platelet parameters in small for gestational age preterm infants
Source: Front Pediatr. 2025 Aug 26;13:1622610. doi: 10.3389/fped.2025.1622610 (PMC12417430; doi:10.3389/fped.2025.1622610)
Supplement: Supplementary file 1 [file Table1.docx]

| **Table 1 The percentage range of MPV, PLT, and PDW in different group** | | | | | | | |
| --- | --- | --- | --- | --- | --- | --- | --- |
| **MPV** | **P5** | **P10** | **P25** | **P50** | **P75** | **P90** | **P95** |
| ***Control group*** |  |  |  |  |  |  |  |
| Day1 | 9 | 9.3 | 9.7 | 10.2 | 10.9 | 11.5 | 11.7 |
| Day2 | 9.33 | 9.43 | 9.9 | 10.5 | 11.275 | 11.8 | 12.3 |
| Day3 | 9.565 | 9.89 | 10.6 | 11.25 | 11.8 | 12 | 12.4 |
| Day4 | 9.98 | 10.16 | 10.6 | 10.9 | 11.5 | 12.04 | 12.46 |
| Day5 | 10.34 | 10.38 | 10.5 | 10.9 | 11.3 | 12.02 | 12.06 |
| Day6 | 10.1 | 10.31 | 10.9 | 11.55 | 12.1 | 12.6 | 12.86 |
| Day7 | 10 | 10.3 | 11.1 | 12.1 | 12.7 | 13.1 | 13.25 |
| Day8-12 | 10.7 | 10.89 | 11.325 | 11.85 | 12.4 | 12.9 | 13.11 |
| Day13-16 | 10.46 | 10.6 | 11 | 11.6 | 12.5 | 13.1 | 13.4 |
| Day17-21 | 10.3 | 10.44 | 11 | 11.6 | 12.75 | 13.26 | 13.33 |
| Day22-30 | 10.72 | 10.86 | 11.3 | 11.6 | 12.1 | 12.5 | 12.6 |
| ***PE/E***  ***group*** |  |  |  |  |  |  |  |
| Day1 | 9.1 | 9.2 | 9.4 | 10.1 | 10.6 | 11.3 | 11.6 |
| Day2 | 9.33 | 9.5 | 9.8 | 10.2 | 10.85 | 11.84 | 11.97 |
| Day3 | 9.31 | 9.42 | 10.5 | 10.9 | 11.3 | 12.28 | 12.4 |
| Day4 | 9.94 | 10.1 | 10.4 | 10.7 | 11.2 | 11.6 | 11.98 |
| Day5 | 10.32 | 10.62 | 10.9 | 11.3 | 11.8 | 11.98 | 12.44 |
| Day6 | 10 | 10.15 | 10.825 | 11.25 | 11.925 | 12.7 | 13.425 |
| Day7 | 11.15 | 11.6 | 12.15 | 12.4 | 13.175 | 13.22 | 13.31 |
| Day8-12 | 10.21 | 10.41 | 11.225 | 11.6 | 12.475 | 13 | 13.485 |
| Day13-16 | 10.45 | 10.5 | 11 | 11.8 | 12.55 | 12.9 | 13.1 |
| Day17-21 | 10.33 | 10.64 | 10.95 | 12 | 12.6 | 13.28 | 13.48 |
| Day22-30 | 10.44 | 10.74 | 11 | 11.7 | 12.2 | 13.04 | 13.26 |
| ***Total*** |  |  |  |  |  |  |  |
| Day1 | 9 | 9.2 | 9.7 | 10.1 | 10.8 | 11.5 | 11.7 |
| Day2 | 9.3 | 9.5 | 9.9 | 10.4 | 11.1 | 11.8 | 12.3 |
| Day3 | 9.38 | 9.56 | 10.5 | 11.1 | 11.7 | 12.16 | 12.4 |
| Day4 | 9.89 | 10.1 | 10.45 | 10.8 | 11.375 | 11.74 | 12.505 |
| Day5 | 10.305 | 10.41 | 10.7 | 11.2 | 11.8 | 12 | 12.095 |
| Day6 | 10.015 | 10.16 | 10.825 | 11.45 | 12.1 | 12.6 | 13.255 |
| Day7 | 10.1 | 10.5 | 11.3 | 12.1 | 12.8 | 13.2 | 13.4 |
| Day8-12 | 10.4 | 10.7 | 11.3 | 11.8 | 12.425 | 12.99 | 13.245 |
| Day13-16 | 10.415 | 10.6 | 11 | 11.75 | 12.525 | 13.07 | 13.37 |
| Day17-21 | 10.3 | 10.47 | 11 | 11.85 | 12.7 | 13.3 | 13.415 |
| Day22-30 | 10.465 | 10.83 | 11.225 | 11.7 | 12.2 | 12.85 | 13.035 |

| **PDW** | **P5** | **P10** | **P25** | **P50** | **P75** | **P90** | **P95** |
| --- | --- | --- | --- | --- | --- | --- | --- |
| ***Control group*** |  |  |  |  |  |  |  |
| Day1 | 9.5 | 9.64 | 10.2 | 11.1 | 12.1 | 14.36 | 16.08 |
| Day2 | 9.53 | 9.83 | 10.45 | 12.15 | 13.575 | 14.57 | 15.355 |
| Day3 | 10.09 | 10.46 | 11.325 | 12.85 | 14.275 | 15.86 | 16.87 |
| Day4 | 10.26 | 10.78 | 12.3 | 13.3 | 15.6 | 16.84 | 17.04 |
| Day5 | 10.46 | 10.62 | 10.9 | 12.6 | 14 | 15.5 | 15.9 |
| Day6 | 10.8 | 11.08 | 12.525 | 14.7 | 16.75 | 20.11 | 22 |
| Day7 | 10.9 | 11.53 | 12.5 | 14.75 | 18.9 | 21.78 | 23.015 |
| Day8-12 | 11.4 | 12.1 | 12.95 | 15 | 17.25 | 19.7 | 20.95 |
| Day13-16 | 11.365 | 11.63 | 12.7 | 14.35 | 16.525 | 21.11 | 22.64 |
| Day17-21 | 10.81 | 11.16 | 12.9 | 14.8 | 17.35 | 18.86 | 20.36 |
| Day22-30 | 11.62 | 12 | 12.4 | 13.4 | 16.1 | 20.4 | 21.28 |
| ***PE/E***  ***group*** |  |  |  |  |  |  |  |
| Day1 | 9.005 | 9.3 | 9.975 | 10.9 | 12.4 | 13.8 | 15.44 |
| Day2 | 9.63 | 9.96 | 10.55 | 11.5 | 12.85 | 15.24 | 16.11 |
| Day3 | 8.9 | 8.98 | 11.8 | 12.4 | 13.75 | 15.06 | 15.19 |
| Day4 | 10.04 | 10.26 | 10.6 | 11.9 | 13.6 | 17.48 | 21.34 |
| Day5 | 11.3 | 11.44 | 12.5 | 14.1 | 15.2 | 16.9 | 18.1 |
| Day6 | 10.55 | 10.75 | 11.7 | 13.8 | 16.05 | 21.3 | 22.725 |
| Day7 | 12.93 | 13.56 | 13.875 | 18.85 | 22.25 | 23.35 | 23.575 |
| Day8-12 | 11.3 | 11.43 | 13.15 | 14.65 | 17.65 | 20.58 | 22.055 |
| Day13-16 | 11.5 | 11.7 | 12.35 | 14.8 | 18.3 | 20.5 | 21.1 |
| Day17-21 | 10.84 | 11.36 | 12.25 | 15.3 | 17.1 | 21.38 | 23.08 |
| Day22-30 | 11.2 | 11.66 | 11.9 | 14.6 | 15.7 | 19.66 | 21.94 |
| ***Total*** |  |  |  |  |  |  |  |
| Day1 | 9.2 | 9.5 | 10.2 | 11 | 12.2 | 14 | 15.52 |
| Day2 | 9.6 | 9.9 | 10.5 | 11.5 | 13.5 | 14.6 | 16.2 |
| Day3 | 9.22 | 9.82 | 11.7 | 12.8 | 14.2 | 15.24 | 16.84 |
| Day4 | 10.165 | 10.3 | 11.425 | 12.65 | 14.9 | 16.87 | 18.545 |
| Day5 | 10.71 | 10.94 | 11.85 | 13.55 | 15.075 | 16.2 | 17.25 |
| Day6 | 10.63 | 10.83 | 12 | 14.4 | 16.725 | 21.54 | 22 |
| Day7 | 10.93 | 11.84 | 13.325 | 15.15 | 20.975 | 22.66 | 23.395 |
| Day8-12 | 11.3 | 11.7 | 13.1 | 14.8 | 17.5 | 19.78 | 21.54 |
| Day13-16 | 11.5 | 11.62 | 12.7 | 14.6 | 17.05 | 20.98 | 22.04 |
| Day17-21 | 10.77 | 11.14 | 12.5 | 15 | 17.425 | 19.23 | 22.135 |
| Day22-30 | 11.495 | 11.7 | 12.25 | 13.8 | 16 | 20.7 | 21.75 |

| **PLT** | **P5** | **P10** | **P25** | **P50** | **P75** | **P90** | **P95** |
| --- | --- | --- | --- | --- | --- | --- | --- |
| ***Control group*** |  |  |  |  |  |  |  |
| Day1 | 99.4 | 126.8 | 168.25 | 213 | 258.5 | 311.6 | 340.2 |
| Day2 | 89 | 101 | 144 | 187 | 235 | 282 | 332 |
| Day3 | 59 | 63.8 | 96 | 148 | 190 | 249.6 | 286.6 |
| Day4 | 44.3 | 51.4 | 112 | 149 | 218 | 269.8 | 274.5 |
| Day5 | 50 | 55.5 | 73.25 | 171.5 | 231 | 262 | 276 |
| Day6 | 49.1 | 70.8 | 116.5 | 176 | 245 | 315.4 | 364.3 |
| Day7 | 69.1 | 74.4 | 127 | 181 | 249.5 | 282 | 368.8 |
| Day8-12 | 120 | 148 | 188 | 264 | 355 | 415 | 539 |
| Day13-16 | 164.35 | 185.1 | 244.25 | 340 | 392 | 455.4 | 515.65 |
| Day17-21 | 124.1 | 131.6 | 207.5 | 313 | 402.5 | 556.6 | 591.7 |
| Day22-30 | 122 | 148 | 181 | 289 | 366 | 434.2 | 539.9 |
| ***PE/E***  ***group*** |  |  |  |  |  |  |  |
| Day1 | 104.2 | 125.2 | 148.5 | 193 | 238.5 | 271.4 | 315.1 |
| Day2 | 78 | 105 | 150.5 | 178 | 227 | 262 | 275 |
| Day3 | 67.8 | 81.6 | 117 | 143 | 182 | 260.6 | 280.4 |
| Day4 | 56.5 | 61 | 81.5 | 142 | 183.5 | 226.4 | 233.5 |
| Day5 | 54.35 | 62.4 | 98 | 126.5 | 175 | 235.2 | 252.15 |
| Day6 | 74.1 | 97.2 | 137.25 | 216 | 244.75 | 307 | 322.25 |
| Day7 | 52.85 | 62.1 | 97.25 | 126 | 179.25 | 188.7 | 207.9 |
| Day8-12 | 118.8 | 134.8 | 182.5 | 236.5 | 322.5 | 407.1 | 459.05 |
| Day13-16 | 133.6 | 173.6 | 218 | 278 | 368 | 425.4 | 454.6 |
| Day17-21 | 121.2 | 159.6 | 233 | 312 | 381 | 451.8 | 495.4 |
| Day22-30 | 177.85 | 179.4 | 239.25 | 283.5 | 337 | 367.1 | 410.45 |
| ***Total*** |  |  |  |  |  |  |  |
| Day1 | 100 | 125 | 161 | 207 | 254 | 306 | 334 |
| Day2 | 83.4 | 101.4 | 147 | 182 | 234 | 264.9 | 307.2 |
| Day3 | 60.75 | 66 | 103.25 | 145 | 190.75 | 259.3 | 288.05 |
| Day4 | 48.25 | 58.5 | 93.25 | 143.5 | 207.25 | 258.5 | 273.75 |
| Day5 | 51.9 | 55.7 | 88.25 | 137 | 219 | 251.6 | 258.55 |
| Day6 | 58 | 73 | 126 | 196 | 245 | 309 | 357 |
| Day7 | 55 | 70 | 104 | 162 | 243 | 281 | 367 |
| Day8-12 | 120 | 135 | 186 | 255 | 343.5 | 415 | 483 |
| Day13-16 | 148 | 178 | 241.5 | 309 | 383.5 | 448 | 494 |
| Day17-21 | 116.85 | 133.1 | 208.75 | 312.5 | 400.75 | 510.4 | 580.6 |
| Day22-30 | 148 | 177 | 187 | 289 | 346 | 407.8 | 451 |
