# Supplementary material for: Effects of pre-eclampsia/eclampsia on platelet parameters in small for gestational age preterm infants
Source: Front Pediatr. 2025 Aug 26;13:1622610. doi: 10.3389/fped.2025.1622610 (PMC12417430; doi:10.3389/fped.2025.1622610)
Supplement: Supplementary file 2 [file Table2.docx]

| **Table 2. Results of PDW, PLT, and MPV compared between PE/E and non-PE/E groups** | | | | | | |
| --- | --- | --- | --- | --- | --- | --- |
| **PDW** | No-PE/E group |  |  | PE/E group |  |  |
|  | Mean | SD | N | Mean | SD | N |
| Day1 | 11.598060 | 2.179732 | 155 | 11.387800 | 2.120179 | 82 |
| Day2 | 12.262160 | 2.433651 | 74 | 11.931920 | 2.125733 | 47 |
| Day3 | 13.008820 | 2.117226 | 34 | 12.439130 | 2.290839 | 23 |
| Day4 | 13.641180 | 2.310319 | 17 | 13.252940 | 4.100018 | 17 |
| Day5 | 12.722220 | 2.098677 | 9 | 14.192310 | 2.303064 | 13 |
| Day6 | 15.042860 | 3.513511 | 28 | 14.818750 | 4.398973 | 16 |
| Day7 | 15.968750 | 4.055855 | 32 | 18.330000 | 4.573122 | 10 |
| Day8-12 | 15.443660 | 3.129524 | 71 | 15.530950 | 3.494769 | 42 |
| Day13-16 | 15.215380 | 3.421809 | 52 | 15.496770 | 3.585386 | 31 |
| Day17-21 | 15.214290 | 3.221905 | 35 | 15.373910 | 4.006723 | 23 |
| Day22-30 | 14.782350 | 3.381426 | 17 | 14.905880 | 3.768035 | 17 |

| **PLT** | No-PE/E group |  |  | PE/E group |  |  |
| --- | --- | --- | --- | --- | --- | --- |
|  | Mean | SD | N | Mean | SD | N |
| Day1 | 215.949400 | 72.290710 | 158 | 198.939800 | 61.928630 | 83 |
| Day2 | 191.395100 | 70.742790 | 81 | 181.823500 | 62.594470 | 51 |
| Day3 | 150.488900 | 70.655570 | 45 | 153.703700 | 71.635730 | 27 |
| Day4 | 163.608700 | 84.190120 | 23 | 139.434800 | 71.293780 | 23 |
| Day5 | 156.250000 | 87.859360 | 16 | 141.272700 | 64.599270 | 22 |
| Day6 | 193.384600 | 101.999900 | 39 | 206.818200 | 89.046610 | 22 |
| Day7 | 193.046500 | 99.108880 | 43 | 133.500000 | 59.409100 | 18 |
| Day8-12 | 281.234600 | 131.316600 | 81 | 257.920000 | 107.789800 | 20 |
| Day13-16 | 331.500000 | 114.694300 | 54 | 293.000000 | 102.638500 | 37 |
| Day17-21 | 319.837200 | 156.921400 | 43 | 311.560000 | 121.206800 | 25 |
| Day22-30 | 289.894700 | 139.819700 | 19 | 283.777800 | 74.955320 | 18 |

| **MPV** | No-PE/E group |  |  | PE/E group |  |  |
| --- | --- | --- | --- | --- | --- | --- |
|  | Mean | SD | N | Mean | SD | N |
| Day1 | 10.339350 | 0.8520027 | 155 | 10.118520 | 0.8442616 | 81 |
| Day2 | 10.621620 | 0.9260714 | 74 | 10.365960 | 0.8382893 | 47 |
| Day3 | 11.126470 | 0.8670077 | 34 | 10.869570 | 0.9725481 | 23 |
| Day4 | 11.041180 | 0.8185802 | 17 | 10.882350 | 0.8924932 | 17 |
| Day5 | 11.033330 | 0.6614378 | 9 | 11.392310 | 0.7983958 | 13 |
| Day6 | 11.517860 | 0.9193611 | 28 | 11.425000 | 1.063015 | 16 |
| Day7 | 11.819360 | 1.077781 | 31 | 12.450000 | 0.831665 | 10 |
| Day8-12 | 11.855710 | 0.7895395 | 70 | 11.795240 | 0.9682654 | 42 |
| Day13-16 | 11.798110 | 0.9422744 | 53 | 11.780650 | 0.9751647 | 31 |
| Day17-21 | 11.808570 | 1.070561 | 35 | 11.839130 | 1.113446 | 23 |
| Day22-30 | 11.694120 | 0.6841698 | 17 | 11.805880 | 0.9724136 | 17 |
| PE/E, preeclampsia/eclampsia; SD standard deviation; PLT platelet coun; MPV mean platelet volume; PDW platelet distribution width | | | | | | |
